# Does Perceptual-Motor Calibration Generalize across Two Different Forms of Locomotion? Investigations of Walking and Wheelchairs

**DOI:** 10.1371/journal.pone.0054446

**Published:** 2013-02-12

**Authors:** Benjamin R. Kunz, Sarah H. Creem-Regehr, William B. Thompson

**Affiliations:** 1 Department of Psychology, University of Dayton, Dayton, Ohio, United States of America; 2 Department of Psychology, University of Utah, Salt Lake City, Utah, United States of America; 3 School of Computing, University of Utah, Salt Lake City, Utah, United States of America; Bielefeld University, Germany

## Abstract

The relationship between biomechanical action and perception of self-motion during walking is typically consistent and well-learned but also adaptable. This perceptual-motor coupling can be recalibrated by creating a mismatch between the visual information for self-motion and walking speed. Perceptual-motor recalibration of locomotion has been demonstrated through effects on subsequent walking without vision, showing that learned perceptual-motor coupling influences a dynamic representation of one's spatial position during walking. Our present studies test whether recalibration of wheelchair locomotion, a novel form of locomotion for typically walking individuals, similarly influences subsequent wheelchair locomotion. Furthermore, we test whether adaptation to the pairing of visual information for self-motion during one form of locomotion transfers to a different locomotion modality. We find strong effects of perceptual-motor recalibration for matched locomotion modalities – walking/walking and wheeling/wheeling. Transfer across incongruent locomotion modalities showed weak recalibration effects. The results have implications both for theories of perceptual-motor calibration mechanisms and their effects on spatial orientation, as well as for practical applications in training and rehabilitation.

## Introduction

Although walking is an action that many take for granted, there are complex perceptual, cognitive and motor components involved in this well-learned, everyday action. Walking requires that we view our surroundings, establish a goal location or object, and then coordinate a series of precise motor movements to travel towards that object or location. That we normally walk accurately to our target location suggests that our spatial perception and motor movements are tightly coordinated. This coordination is adaptable, as evidenced by the ability to alter one’s actions in response to changes in the external environment – one easily changes stride to walk up a steep hill or when walking into a driving wind.

While walking, the visual information about the rate of self-movement typically matches the biomechanical indicators of self-movement. In other words, the world appears to move by at the same speed as walking. For common, well-practiced tasks like walking, this relationship between perceptual-indicators of self-movement and the biomechanical actions involved in locomotion is probably well-learned. A number of studies, however, have supported the notion that the relationship between perceived self-motion and biomechanical action is flexible and can be adapted. In these experiments, a novel pairing between visual information about the rate of self-motion and the movements involved in locomotion influences subsequent locomotion. For example, participants adapt to a mismatch between visual information for self-movement and biomechanical information for self-movement. Following this adaptation, participants perform visually-*directed* (or open-loop) actions such as walking to a previously-viewed target while blindfolded. These blind-walking tasks rely on a perceptual representation to guide actions in the absence of continuous visual feedback [1], [2], [3] and are a commonly-used measure of distance perception. Whereas blind-walking performance under normal circumstances is generally accurate, the distances walked while blindfolded are underestimated after adaptation to a rate of visual flow that is faster than the biomechanical rate of walking. Similarly, after adaptation to a rate of visual flow slower than the rate of walking, blind-walking performance reflects an overshoot of targets [4], [5] (or see [6], [7] for accounts of non-visual perceptual-motor recalibration). The recalibration of rotations also influences subsequent turning in-place behavior [4], [8], [9].

The effect of locomotion adaptation on blind-walking and imagined walking [10] is robust. However, there are unanswered questions about how this adaptation influences perceptually-guided actions more broadly. There is some inconsistency in current findings concerning how forms of locomotion other than walking may be calibrated. In a series of experiments Rieser and colleagues [4] demonstrated that recalibration of locomotion influenced subsequent blind-walking to targets, whether it was performed via forward walking or side-stepping. Blind-throwing and blind-rotations were not influenced by recalibration of locomotion (see also [11]). From these results, they conclude that recalibration of locomotion is not limb-specific (i.e. doesn’t exclusively recalibrate forward walking leg movements) but influences actions that are functionally equivalent to walking. In other words, recalibration of walking should influence other actions that serve to move the viewer through the environment. Others have shown that recalibration is limb-specific and does not influence motor movements that do not involve the recalibrated limb [12] or that the influence of recalibration of walking on other forms of functionally related locomotion, such as sidestepping [7] or crawling [13] is weak or more variable.

Our present studies test the generalizability of locomotor recalibration using walking and wheelchair locomotion. More specifically, we attempt to evaluate two accounts of perceptual-motor recalibration of locomotion: a limb-specific account and more general recalibration of functionally-related actions. First, we ask whether established effects of perceptual-motor recalibration on walking extend to wheelchair locomotion, a novel form of locomotion for walking individuals. Wheelchairs as a locomotion modality are useful to study because they serve the same function as walking – to translate through space – but they require very different limb movement (arms versus legs). Second, we test whether adaptation to the pairing of visual information for self-motion during one form of locomotion transfers to a different locomotion modality – these experiments, in particular, are an attempt to distinguish between the two accounts of recalibration of locomotion. Across four experiments we used a methodology of adaptation relying on virtual environment head-mounted-display technology where the visual information for self movement could be decoupled from biomechanical information for self movement. Perceptual-motor information for two types of locomotion – walking and wheelchair locomotion – were manipulated. Blind-walking and blind-wheeling were measured.

Our results are suggestive of a functional organization of perception and action [4] in which the perceptual-motor experience of locomotion calibrates matched open-loop locomotion (e.g. walking or wheeling without vision). However, we show only a weak influence of recalibration of walking on a biomechanically distinct action that serves the same functional goal. Given this weak influence of recalibration of walking on subsequent wheeling and no effect of recalibration of wheeling on walking, we discuss some modifications to the functional account of perceptual-motor recalibration. More specifically, we suggest that the generalizability of perceptual-motor calibration of walking is not characterized by a broadly-defined functional similarity between walking and other forms of locomotion, but is moderated by the biomechanical similarity and familiarity of actions other than walking.

## Experiment 1

Experiment 1 served two purposes. First, we establish a virtual environment methodology used to test the effects of perceptual-motor calibration of walking on subsequent blind-walking, as seen in previous experiments in real [4] and virtual [14], [15], [16] environments. Second, our results serve as a baseline for comparison with Experiments 2–4 in which the type of locomotion and response measure were varied. Consistent with both accounts of recalibration of locomotion, we predicted that visual-motor adaptation during walking would influence subsequent blind-walking performance. More specifically, we anticipated that adaptation to a rate of visual flow that is slower than walking speed would produce an increase in blind-walked distances while adaptation to a rate of visual flow faster than walking speed would yield a decrease in distances walked.

### Methods

#### Ethics Statement

All experiments were approved by the University of Utah Institutional Review Board. Written consent was obtained from all participants prior to their participation.

#### Participants

A total of 21 participants were randomly assigned to either a visually slower condition (*n* = 11), in which the visual information about self-motion was half the participants’ actual walking speeds, or visually faster intervention condition (*n* = 10), in which the visual speed was twice the walking speed. Participants were screened for normal visual acuity and stereo vision. Each participant was run through the experiment individually over the course of approximately one hour.

#### Materials

We used a virtual environment during the intervention phase to present decoupled visual flow information and biomechanical information for self-motion while participants moved through the environment. An immersive virtual environment (VE) was presented to participants using an NVIS nVisor SX head-mounted display (HMD) with a 1280×1024 resolution, 60 Hz refresh rate, horizontal and vertical field of view of approximately 42 and 34 degrees, respectively, and 100% stereo overlap between the two eyes. HMD optical pincushion distortion was corrected using a GPU shader program, without introducing additional latency (see [17] for an explanation of HMD calibration and correction).

Three degree-of-freedom positional tracking was done optically using a Worldviz PPT-H camera-based tracking system, and three degree-of-freedom orientation tracking was done with a combination of optical tracking for yaw using the PPT-H system and accelerometers for pitch and roll using an Intersense IC3 orientation sensor. The eye height of the virtual viewpoint was scaled to each participant’s eye-height, using the position of the optical markers on the HMD. A *Windows XP* computer running *Worldviz PPT Studio* integrated the results into a single 6 DOF indication of HMD position.

**Figure 1:**
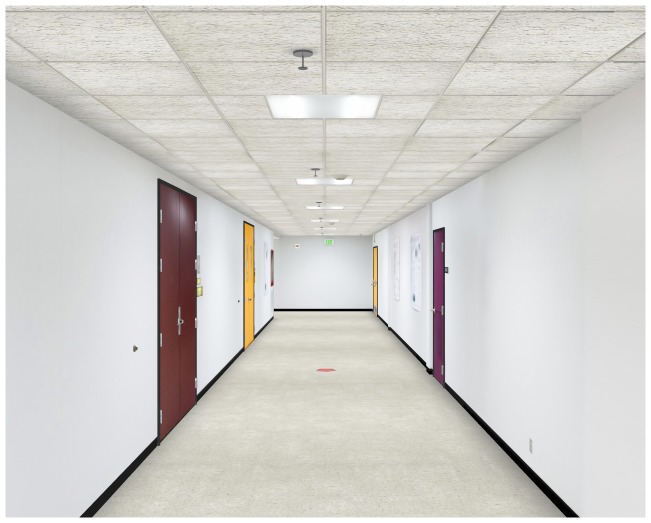
Virtual environment hallway.

A second Windows XP computer running *Worldviz Vizard* (version 3.16) software rendered the virtual environment. The virtual environment consisted of a hallway measuring 3 m×12 m, modeled after a real-world hallway (see Figure 1). In the visually-slower condition, participants’ locomotion along the length of the hallway was scaled so that the coordinates from the head tracker were multiplied by a gain factor of 0.5. In the visually faster condition, a gain change factor of 2 was applied. The gain factor was only applied to the horizontal plane, in the direction of the hallway, so that movements in any other direction (participants were discouraged from moving from side-to-side except for normal sway in forward walking) were not scaled (see also [18], [19]). Rotations in yaw, pitch and roll were not scaled.

Real world blind-walking training and pretest/posttest trials were conducted in a 3.15 m wide by approximately 25 m long hallway. To obscure the tiled floor texture, a gray carpet was rolled out onto the hallway floor, extending in width from wall to wall and 9.5 m down the length of the hallway. Targets consisting of six colored foam-board shapes were presented on the floor of the hallway at randomized distances of 3.5, 4.5 or 5.5 m. Distances walked were measured using a *Sonin Multi-Measure Combo Pro*, ultrasonic electronic measuring tape.

Noise-canceling headphones and an mp3 player playing pink noise were used to reduce sound localization as a cue to position tracking, and a wireless microphone was used to avoid acoustic localization of the experimenter. Participants wore a *Mindfold* blindfold that could easily be raised and lowered over the eyes during the experiment and training.

#### Design

For each participant, the experiment consisted of four phases. The first phase involved training and instruction. Training was immediately followed by the pretest phase, in which participants performed blind-walking trials in the real world hallway. The third phase was the adaptation intervention, in which participants moved through a virtual hallway for a period of approximately 5 to 7 minutes while experiencing visual flow that differed from participants’ physical movement speed. Half of the participants experienced visual flow rates faster (two times) than movement speed, while the other half experienced visual flow rates slower (half) their movement speeds. This was immediately followed by the posttest blind-walking phase in the real world hallway. This within-subject design allows for comparisons of performance prior to and after the intervention. Visual flow rate was a between-subject variable. There was no “matched” flow condition, as it is unknown whether the perceived speed of self-motion using an HMD matches the perceived speed of self-motion in the real world. The value of a “matched” condition is limited without a more comprehensive investigation of speed perception under these particular visual and biomechanical conditions, and such a study is beyond the scope of the current paper. Moreover, while a “matched” condition was used in some of our previous work [5], [20], critical to our present design was the ability to assess whether within-subject pre/post difference resulted in a given adaptation condition *and* whether between-subject flow manipulations led to differences in the magnitude and direction of the recalibration effect.

#### Procedure

After providing informed consent, participants were tested for visual acuity. Participants unable to read an eye chart at 20/30 or better or who demonstrated lack of stereo fusion were excluded from analysis. After reading written instructions for the task, participants were outfitted with noise-canceling headphones and a blindfold and were informed as to the general nature of the task to be completed. Participants then practiced walking without vision in the hallway, first being led by an experimenter by the shoulders and then walking independently with an experimenter following behind and providing verbal indications of when to turn and stop. After approximately 5 minutes of blind-walking, or until the participant demonstrated and professed comfort with blind-walking, the experimenter verbally described and demonstrated the experimental task.

Participants were instructed to view a target at a given distance and to form a “good image” of the target and the surroundings. A “good image” was defined as one that, once obtained, would allow the participant to visualize the environment and target after his/her eyes were closed. They were instructed to lower the blindfold and walk to the target location, updating their position as they walked. The experimenter demonstrated the task before beginning the experiment trials.

Following training, participants were turned to face the opposite direction in the real world hallway and were positioned so that they stood on the edge of the carpet for the pretest blind-walking trials. The pretest consisted of 10 trials, including an initial practice trial that was excluded from analysis. The initial practice trial (target presented at 4.5 m) was intended only to ensure that the participant understood the task. The participant was unaware that it was a practice trial and no feedback was given to the participant regarding blind-walking accuracy). During the nine experimental trials, targets appeared at distances of 3.5, 4.5 and 5.5 m, each repeated three times in random order. On each trial, participants viewed the target and surroundings as long as necessary to form a good mental image. They then lowered the blindfold and walked to the target location. A second experimenter removed the target before the participant reached its location. The walked distance was measured and recorded on each trial and the participant was led back to the starting location while blindfolded via an indirect route to obscure the distance walked. No feedback regarding performance was given until after the experiment.

Following the 10th trial, participants were led into the adjacent virtual environment lab while blindfolded to begin the intervention phase. Once inside the lab, participants were outfitted with the HMD without viewing the physical lab space. Once the HMD was fitted, participants viewed a virtual hallway and were instructed to walk through the virtual hallway with their eyes open and to stop walking upon hearing a tone. Once the participants stopped, the experimenter instructed them to close their eyes (the HMD screen was blanked) and the experimenter guided them back to the physical starting location via an indirect route. During this intervention phase, depending upon condition, participants viewed the virtual hallway moving by at a rate either half their walking speed (visually slower condition) or twice their walking speed (visually faster condition). On each trial, a tone signaling the participant to stop walking was played at a physically walked distance of 5, 6, or 7 m, with each distance repeated five times in random order for a total of 15 trials. During this phase, although the physical starting point remained the same, participants viewed one of three different starting points in the virtual hallway (randomly selected from one of three offsets along the participants’ heading direction). The varied starting points were intended to reduce the memorization of distances walked during the intervention stage and to encourage the participants to look around while walking with eyes open.

Following the 15th and final trial of the intervention phase, participants were blindfolded and led back to the adjacent hallway to complete the posttest. During posttest, participants completed nine more trials of blind-walking to targets, using the same randomized target and distance order employed in the pretest.

### Results

**Figure 2:**
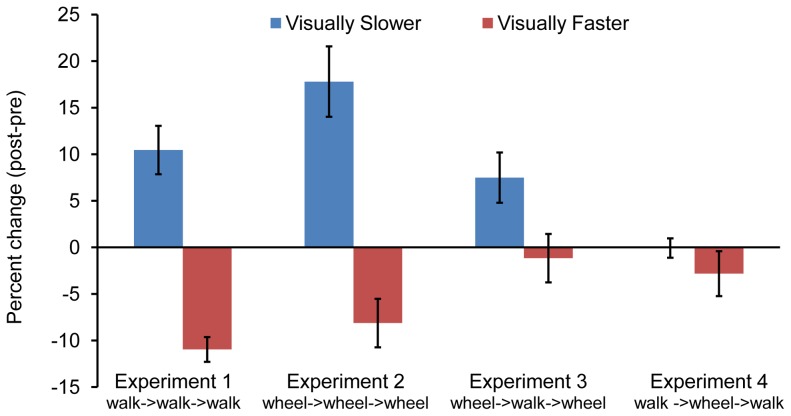
Percent change in distance walked (Experiments 1 and 4) and wheeled (Experiments 2 and 3).

Distances walked were scaled by actual target distances to obtain a percent distance walked for each trial. These values were averaged for each target distance (mean percent walked for each target distance). When averaged across target distance, participants in the visually slower condition walked an average of 10.45% (*SD*  = 8.61) farther in the posttest than in the pretest, whereas participants in the visually faster condition walked 10.96% (*SD*  = 4.20) shorter in the posttest, compared to pretest (see Figure 2).

A 2 (testing session: pretest or posttest) ×3 (distance) ×2 (visual speed condition) repeated measures ANOVA was performed on the mean percent walked. It revealed a significant interaction between testing session (pre vs. post) and condition, *F*(1,19)  = 50.70, *p*<.001, η_p_^2^  = .73. This interaction indicates that the change in distance walked from pre- to post-test differed as a function of the visual-flow speed manipulation. This difference was confirmed by an independent samples t-test performed on percent change (pre- to post-test) comparing the visually slower and visually faster conditions, *t*(19)  = 7.12, *p*<.001, *d* = 3.16. Follow-up paired samples *t*-tests indicated significant differences between pretest and posttest percent walked for the visually-slower condition, *t*(10)  = −4.03, *p* = .002, *d* = 1.21 and the visually-faster condition, *t*(9) = 8.264, *p*<.001, *d* = 2.6, with effects in opposite directions.

Furthermore, there was a significant main effect of target distance, *F*(2,38)  = 34.62, *p*<.001, η_p_^2^  = .65. Planned contrasts indicated that the percent of the distance walked was less for 3.5 m targets versus 4.5 m targets, *F*(1,19)  = 15.87, *p* = .001, η_p_^2^  = .46 and less for 4.5 m targets than for 5.5 m targets, *F*(1,19)  = 20.66, *p*<.001, η_p_^2^  = .52. Despite a tendency to underestimate the near distance (3.5 m) and overestimate the far distance (5.5 m), pretest blind-walking was generally accurate, as has been shown in previous studies employing blind-walking as a measure of perceived distance (see Table 1). This finding that generally accurate locomotion is influenced by perceptual-motor recalibration is apparent in several of the studies that follow.

**Table 1:** Slope, intercept and R^2^ for linear fit through the mean values walked or wheeled for each target distance.

Experiment	Pre/ Posttest Task	Recalibration	y- intercept	Slope	R^2^
1	Walking	Walking	−1.179	1.280	1.000
2	Wheeling	Wheeling	−0.712	1.182	0.999
3	Wheeling	Walking	−0.432	1.105	0.998
4	Walking	Wheeling	−0.927	1.214	0.999

**Figure 3:**
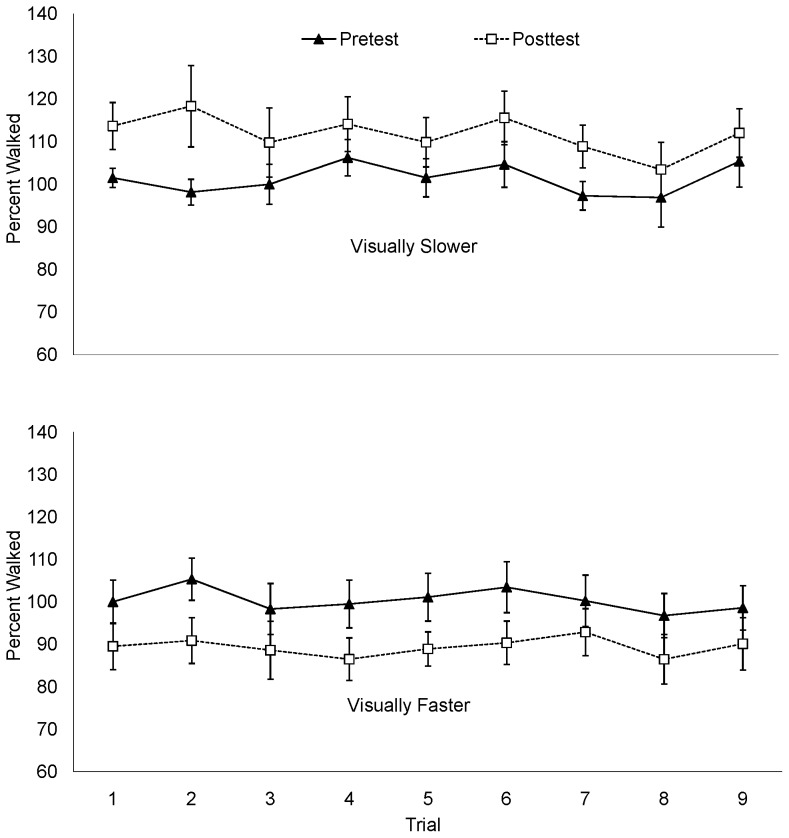
Percent walked by trial in Experiment 1.

The mean percent of the actual target distance is plotted by trial in Figure 3. There was no apparent tendency to walk farther as the experiment progressed. To determine whether the recalibration effect was apparent immediately following the intervention phase, the difference between the percent distance walked on the first posttest trial and the mean pretest percent distance walked was calculated. Consistent with the mean posttest data, the distance walked in the first posttest trial of the visually-slower condition increased 12.4% compared to mean pretest percent walked; a paired samples t-test confirmed a significant difference between the first posttest trial and the mean pretest distances walked, *t*(10)  = −3.93, *p* = .003. The distance walked in the first posttest trial of the visually-faster condition decreased significantly by 10.79%, *t*(10)  = 4.61, *p* = .001. An independent samples *t*-test conducted on the percent change (first posttest percent walked – mean pretest percent walked) showed a significant difference between the visually-faster and visually-slower conditions, *t*(19)  = 5.8, *p*<001, *d* = 2.56.

### Discussion

These results are qualitatively similar to previous experiments assessing the influence of recalibration of walking on blind-walking accuracy [4], [5]. This is one of the few recalibration experiments to utilize natural walking in an HMD-based VE in order to produce visual-motor recalibration, and to demonstrate the effect of HMD-based recalibration on subsequent real-world locomotion (see also [20]). Blind-walking performance changed as a function of the rate of visual flow.

It is important to note that several previous experiments employed treadmill walking during the intervention phase, as opposed to naturalistic walking on the ground. This may partly account for an asymmetry in the magnitude of recalibration seen in treadmill recalibration studies, but absent in this experiment. As Durgin et al. [7] have suggested, treadmill walkers likely received haptic, vestibular, kinesthetic, or visual cues that they were immobile with respect to the treadmill – a sort of extreme version of the visually-slower condition. In other words, while visual flow specified a rate of movement through space, they simultaneously received information from the visible stationary treadmill handrail or platform that they were not moving at all. HMDs alleviate some of this conflicting information because there is no need to hold a treadmill handrail and no view of the stationary surrounding environment while walking. Moreover, because the present experiment created a visual-motor mismatch while walking wearing an HMD, the vestibular cues matched the kinesthetic information for forward walking. In Rieser and colleagues’ recalibration experiment [4], participants walked at a constant rate of speed on a treadmill that was pulled by a tractor at a different speed, thereby producing a mismatch between motor movements involved in walking and vestibular information about accelerations and decelerations. This experiment also differs from other visual feedback manipulations of HMD locomotion [15], [16], [20] in that there was no goal-directed walking during the recalibration phase. In other words, participants did not walk to target location and then stop. Instead, they walked at a comfortable pace with no goal location and stopped upon hearing an auditory tone. In this way, the present design is more akin to previous recalibration experiments employing treadmill-based adaptation [4], [21] in which participants simply walked without the expressed goal of reaching a target location.

## Experiment 2

The results of Experiment 1 are consistent with previous studies of the recalibration of locomotion. Because walking is a primary means of moving through the environment, it is highly adaptable to changing environmental conditions and varied terrain. The perceptual-motor recalibration seen in Experiment 1 is consistent with this adaptability. It is less clear, however, whether unfamiliar or unpracticed forms of movement illustrate a similar flexibility.

Wheelchair locomotion, for example, is functionally similar to walking in that it serves to move one through the environment, but it does so without any involvement of the legs. Consequently, wheelchair locomotion is biomechanically very different from walking in terms of both the effectors used and the rhythm of movement, requiring simultaneous arm movements to turn both wheels the same about in order to move straight ahead. For most individuals, wheelchair locomotion requires an unfamiliar but easily learned sequence of actions. Experiment 2 investigated whether a largely unfamiliar action showed evidence of perceptual-motor calibration analogous to the recalibration of walking. More specifically, to determine whether the perceptual-motor calibration of locomotion could be calibrated via wheelchair locomotion, we assessed its influence on subsequent wheeling. Here again, both accounts of perceptual-motor recalibration would suggest that recalibration of wheelchair locomotion would influence subsequent wheeling behavior because both the adapted action and the subsequent form of locomotion are biomechanically and functionally identical.

### Methods

#### Participants

A total of 20 participants were randomly assigned to either a visually slower (*n* = 10) or visually faster (*n* = 10) recalibration condition. All participants were able to walk without assistance and had little to no experience using wheelchairs.

#### Materials and Design

The same targets, target distances, noise-canceling equipment, blindfold, and real and virtual environments used in Experiments 1 were used in Experiment 2. An *Invacare Tracer EX2* wheelchair was employed during pretest/posttest and the adaptation phase in the virtual world. The wheelchair was modified to keep the orientation of the front wheel in the forward direction, thus restricting the user’s ability to change direction.

#### Procedure

The general procedure used in Experiment 2 closely followed the procedure described in Experiment 1, with notable differences in the means of locomotion in pretest/posttest and intervention phase. Following initial screening procedures, participants practiced using the wheelchair in order to establish a consistent, comfortable wheeling speed. Participants first practiced wheeling with eyes open to locations in the hallway. Participants were instructed to wheel until their feet were lined up with target locations (randomly selected landmarks in the hallway) and to view the hallway as they wheeled. After approximately 7 to 10 minutes of eyes-open wheeling, participants practiced eyes-closed wheeling for about 3 minutes and were given instructions regarding spatial updating during blindfolded wheeling. The experimenter then verbally described and demonstrated the experimental task.

Participants were seated in the wheelchair and were instructed to view a target at a given distance and to form a “good image” of the target and the surroundings, and then to lower the blindfold and wheel themselves to the target location while updating their position as they wheeled. Participants were told to stop the wheelchair when their feet (on the footrests of the wheelchair) were directly over the target. As in Experiment 1, targets were presented at the same distances, in randomized order. The distance wheeled (measured to the participant’s feet after wheeling to the judged target location) was measured and recorded on each trial and the participant was wheeled backwards to the starting location while blindfolded. No feedback regarding performance was given until after the experiment.

During the intervention phase, participants *wheeled* with eyes open through the virtual hallway described in Experiment 1. Depending on condition, participants viewed the virtual hallway moving by at a rate either half their wheeling speed (visually slower condition) or twice their wheeling speed (visually faster condition). Following the 15th and final trial of the intervention phase, participants were blindfolded and wheeled by the experimenter to the real hallway to complete the posttest, consisting of nine trials of blindfolded wheeling to targets.

### Results

Participants in the visually slower condition wheeled an average of 17.8% (*SD*  = 11.96) farther in the posttest than in the pretest, whereas participants in the visually faster condition wheeled 8.13% (*SD*  = 8.24) shorter in the posttest, compared to pretest (see Figure 2).

A 2 (testing session: pretest or posttest) ×3 (distance) ×2 (visual speed condition) repeated measures ANOVA revealed significant main effects of target distance, *F*(2,36)  = 12.17, *p* <.001, η_p_^2^  = .40, testing session, *F*(1,18)  = 4.44, *p* = .049, η_p_^2^  = .20, condition, *F*(1,18)  = 4.76, *p* = .043, η_p_^2^  = .21, an interaction between target distance and condition, *F*(2,36)  = 8.00, *p* = .001, η_p_^2^  = .31. Importantly, as in Experiment 1, we found an interaction between testing session and condition, *F*(1,18)  = 31.87, *p* <.001, η_p_^2^  = .64, revealing that there was a significant difference in the percent change from pre- to post-test between the visually faster and visually slower conditions. This difference was confirmed by an independent samples t-test performed on percent change (pre- to post-test) comparing the visually slower and visually faster conditions, *t*(18)  = 5.65, *p*<.001, *d* = 2.52. Follow-up paired samples *t*- tests indicated significant differences between pretest and posttest percent wheeled for the visually-slower condition, *t*(9)  = −4.71, *p* = .001, *d* = 1.48 and the visually-faster condition, *t*(9)  = 3.12, *p* = .012, *d* = .99, with effects in opposite directions. The main effect of target distance was driven by a significant difference in percent wheeled between targets at 3.5 and 4.5 m, *F*(1,18)  = 16.48, *p* = .001, η_p_^2^  = .48, according to planned contrasts. Across both conditions, pretest blind-wheeling was initially underestimated, but increased over the course of the pretest (see Table 1).

**Figure 4:**
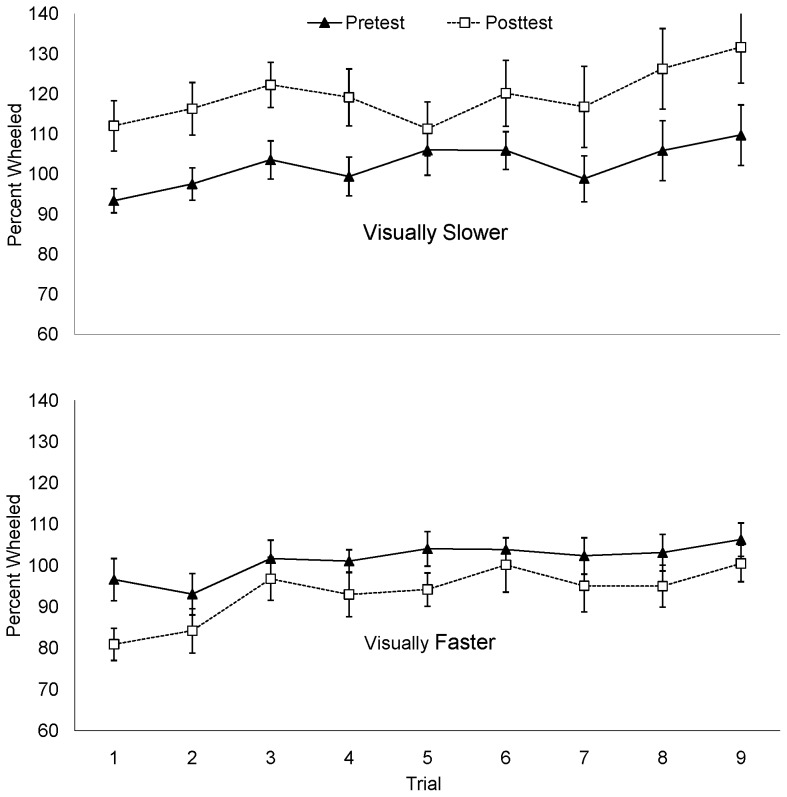
Percent wheeled by trial in Experiment 2.

The mean percent of the actual target distance is plotted by trial in Figure 4. A positive correlation between trial number and the percent distance wheeled averaged across conditions, (*r = *.20, *p*<.001) suggests that distance wheeled increased over successive trials. To determine whether the recalibration effect was apparent immediately following the intervention phase, the difference between the percent of the distance wheeled on the first posttest trial and the mean pretest distance wheeled was calculated. Consistent with the mean posttest data above, the distance wheeled in the first posttest trial of the visually-slower condition increased 9.8% compared to mean pretest percent walked, while the distance walked in the first posttest trial of the visually-faster condition decreased 20.51%. Paired samples *t*-test conducted on the percent change between the first posttest trial and the mean of the pretest trials approached significance for the visually-slower condition, *t*(9)  = −2.23, *p* = .053 and reached significance for the visually-faster condition, *t*(9)  = 5.67, *p*<.001. An independent samples *t*-test conducted on the percent change (first posttest percent walked – mean pretest percent walked) revealed a significant difference between the visually-faster and visually-slower conditions, *t*(18)  = 5.32, *p*<.001, *d* = 2.38. In light of the data from mean pretest and posttest data, these additional analyses suggest that a wheeling practice effect may have amplified an initial change from pretest to the first posttest trial (9.8%) in the visually slower condition but diminished an initially large difference between pretest the first posttest trial (20.51%) in the visually faster condition.

### Discussion

The results from Experiment 2 are similar to those from Experiment 1 and previous recalibration of locomotion experiments [4], [5], [15], [16], though with a larger recalibration effect in the visually slower condition than in the visually faster condition (see Table 2). Given these results, it appears that participants wheeled to previously viewed targets based upon the perceptual-motor relationship learned during the adaptation period. In the visually-slower condition, participants showed an overshoot in wheeling distance in the posttest compared to the pretest; in the visually-faster condition, they showed an undershoot in wheeling distance. This effect was smaller in magnitude than in the visually-slower condition. We attribute this to participants’ tendency to wheel farther as the experiment progressed, regardless of condition. This tendency to wheel farther would obscure the typical undershooting seen in the visually-faster condition (and as seen in Experiment 1). This practice effect would bring posttest wheeling closer to accurate in the visually-faster condition (counteracting the effect of recalibration), but should create a larger spread between pretest and posttest in the visually-slower condition as the experiment progressed.

**Table 2:** Summary of results from Experiments 1–4 with real world pretest and posttest.

Experiment	Condition	Pretest Accuracy	Posttest Accuracy
1. Walk->Walk->Walk			
	Visually Slower	101.31%	111.77%
	Visually Faster	98.62%	89.39%
2. Wheel->Wheel->Wheel			
	Visually Slower	102.24%	120.04%
	Visually Faster	101.42%	93.30%
3. Wheel->Walk->Wheel			
	Visually Slower	100.66%	108.15%
	Visually Faster	100.23%	99.07%
4. Walk->Wheel->Walk			
	Visually Slower	99.14%	99.06%
	Visually Faster	101.04%	98.22%

## Experiment 3

Experiment 1 demonstrated the influence of visual-motor recalibration on subsequent blind-walking performance while Experiment 2 illustrated a novel analogous influence of visual-motor recalibration on subsequent blind-wheeling performance. Given that both familiar and unfamiliar forms of locomotion can be recalibrated when there is a match between the locomotion adaptation and the response, we sought to determine whether the demonstrated recalibration effects are specific to the mode of locomotion used during adaptation, or whether they generalize to other locomotion modalities that serve the same functional goal of translating through the environment.

In Experiment 3 we attempt to determine whether perceptual-motor calibration is functionally defined or biomechanically specific by assessing the influence of recalibration of walking on subsequent wheeling. If the influence of walking adaptation is specific to the calibration of walking, there should be no effect of recalibration on wheelchair locomotion. This finding would support the hypothesis that perceptual-motor calibration is limb-specific [7], [12]. On the other hand, if perceptual-motor calibration is functionally organized so that actions with similar functional goals rely on the learned perceptual-motor coupling, regardless of effectors [4], adaptation in walking should influence subsequent wheelchair locomotion performance.

### Methods

#### Participants

A total of 24 participants were randomly assigned to either a visually slower (*n* = 13) or visually faster (*n* = 11) recalibration condition.

#### Materials and Design

The same targets, target distances, noise-canceling equipment, blindfold, and real and virtual environments used in Experiments 1 and 2 were used in Experiment 3.

#### Procedure

The procedure used in Experiment 3 closely followed the procedure described in Experiment 2, except that the adaptation phase involved walking while viewing discrepant visual flow. As in Experiment 2, participants were trained to use the wheelchair both with eyes open and eyes-closed.

During pretest and posttest, participants were seated in the wheelchair and were instructed to view a target at a given distance and to form a “good image” of the target and the surroundings, and then to lower the blindfold and wheel themselves to the target location while updating their position as they wheeled. Participants were told to stop the wheelchair when their feet (on the footrests of the wheelchair) were directly over the target. As in Experiment 2, targets were presented at the same distances, in randomized order. The distance wheeled (measure to the participant’s feet after wheeling to the judged target location) was measured and recorded on each trial and the participant was wheeled backwards to the starting location while blindfolded. No feedback regarding performance was given until after the experiment.

The adaptation phase following the wheeling pretest was identical to that of Experiment 1. Participants *walked* to distances of 5, 6, and 7 m (repeated five times in random order with an auditory tone indicating the end of the distance walked) with eyes open while viewing a rate of visual flow either half their walking speeds (visually slower condition) or twice their walking speeds (visually faster condition). Following the adaptation phase, participants were blindfolded, guided back to the adjacent hallway and seated in the wheelchair. They then completed the nine posttest blindfolded wheeling trials (using the same random target and target distance order from pretest).

### Results

Participants in the visually slower condition wheeled an average of 7.5% (*SD*  = 9.72) farther in the posttest than in the pretest, whereas participants in the visually faster condition wheeled 1.2% (*SD*  = 8.62) shorter in the posttest, compared to pretest (see Figure 2).

A 2 (testing session: pretest or posttest) ×3 (distance) ×2 (visual speed condition) repeated measures ANOVA performed on mean percent distance wheeled revealed an interaction between testing session and condition, *F*(1, 22)  = .032, *p* = .032, η_p_^2^  = .19, as in Experiments 1 and 2. This interaction reveals that the percent change from pre- to post-test differed significantly between the visually slower and visually faster conditions, confirmed further by an independent samples *t*-test comparing the percent change across visual conditions, *t*(22)  = 2.290, *p* = .034, *d* = .94. Follow-up paired samples *t*-tests indicated significant differences between pretest and posttest percent wheeled for the visually-slower condition, *t*(12)  = −42.78, *p* = .017, *d* = .77, but unlike in Experiment 2, not for the visually-faster condition, *p* = .66. In addition, there was a significant main effect of target distance, *F*(2,44)  = 3.23, *p* = .049, η_p_^2^  = .13. Planned contrasts indicated that the percent wheeled to the 5.5 m target was greater than the percent wheeled to 4.5 m targets, *F*(1,2)  = 5.99, p = .023, η_p_^2^  = .214 but no difference between 3.5 m and 4.5 m, p = .91. Across both conditions, pretest blind-wheeling was typically less than the target distance, but tended to increase over the course of the pretest (see Table 1).

**Figure 5:**
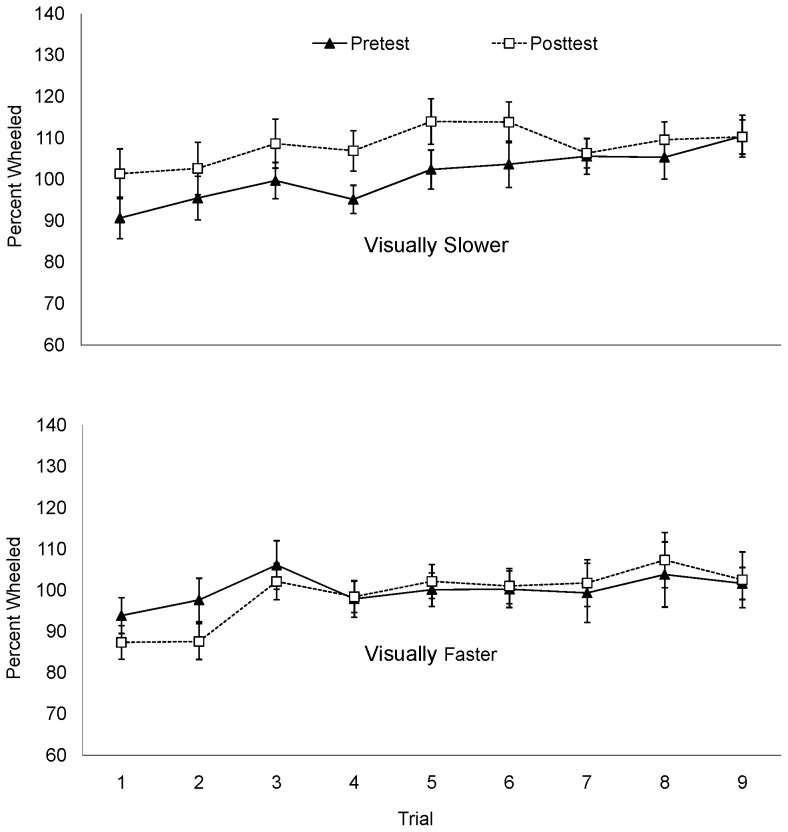
Percent wheeled by trial in Experiment 3.

The mean percent of the target distance wheeled is plotted by trial in Figure 5. As in Experiment 2, a positive correlation between trial number and percent distance wheeled, (*r  = .*19, *p*<.001) suggests that distance wheeled increased over successive trials for both conditions. As in Experiment 2, there was a sizable decrease (12.9%) in distance wheeled from pretest to the first trial of the posttest for the visually-faster condition, *t*(10)  = 3.218, *p* = .009). The first posttest trial in the visually slower condition was not significantly different from the mean pretest distances wheeled (*p* = .92). An independent samples t-test conducted on the difference between the first posttest trial and the mean pretest distance wheeled for each condition was not significant, (*p* = .10). This suggests that the overall difference between conditions was not apparent immediately following the recalibration phase.

### Discussion

Experiment 3 suggests that visual-motor calibration weakly transfers across two types of locomotion. While there remained an overall difference between the visually faster and visually slower conditions, this effect is driven by the pre/post-test difference in the visually slower condition, as there was not a significant pre/post-test change for the visually faster condition. The small difference in the visually faster condition may be, in part, the result of a tendency to wheel farther as the experiment progressed, regardless of condition. As in Experiment 2, it appears that there was initially an effect of recalibration in the visually faster condition but that a practice effect gradually reduced the typical undershoot over repeated trials (i.e. bringing posttest distance wheeled back up to accurate pretest levels). This same practice effect likely increased the mean of pretest distances wheeled in the visually slower condition and could help to explain the small difference between the mean pretest and the first posttest trial (increasing only.7% compared to pretest). Nevertheless, there was, on average, an increase of 7.5% in distance wheeled in the posttest of the visually-slower condition. While the first posttest trial data is informative about an immediate recalibration effect, it is equally important to consider the average posttest data given the limitations of comparing single trial data to a mean pretest score which was influenced by trials over time.

Despite the significant difference between the average percent change in the visually faster and visually slower conditions, the magnitude of the recalibration effect is smaller than would be expected if wheelchair locomotion relied on a perceptual-motor calibration from walking in the same way that walking relies on such a calibration (see Table 2). This smaller effect of calibration on biomechanically distinct, but functionally related, forms of locomotion is consistent with previous studies using different types of locomotion with the legs. Durgin et al. [7] assessed the influence of treadmill walking without vision (which produces subsequent blind-walking overshoot comparable to the visually-slower condition used here) on sidestepping-to-target accuracy. After 1 minute of normal (i.e., forward-facing) treadmill adaptation, there was no effect on sidestepping. Although 5 minutes of treadmill forward walking did produce an overshoot in subsequent sidestepping to targets, the change was more variable than for subsequent forward walking (i.e. nearly half of the participants undershot targets during subsequent side-stepping). The authors also point out that the effect of recalibration of walking on sidestepping in previous studies [4] was of smaller magnitude than the effect on forward walking. Moreover, Withagen and Michaels [13] showed that recalibration of walking influenced subsequent crawling, but this recalibration resulted in target overshoots in both a visually-slower and visually-faster condition.

The present results add to these findings by testing transfer of calibration to locomotion controlled by entirely different limbs, i.e., leg-to-arm movements, but with the same functional goal of translation through space. We show some evidence for recalibration, based on the significant difference in the pre and post test differences between the two visual flow conditions, but the effect is difficult to interpret given that it occurs in a single direction (overshoot for visually slower but no difference for visually faster). However, if there was no influence of recalibration at all, we would expect to see no difference between the visually faster and visually slower conditions. This suggests that there are additional variables beyond a broadly defined *functional similarity* that influence the generalizability of recalibration of locomotion on subsequent actions. To further evaluate the influence recalibration of locomotion on functionally-similar (but biomechanically distinct) actions, we paired wheeling adaptation with a walking response in Experiment 4.

## Experiment 4

Experiment 4 further examines the question of functional generalizability of recalibration by using wheelchair locomotion as the adapted action. More specifically, we investigate whether adaptation to wheeling will influence subsequent walking. Again, a functional organization model of perceptual-motor recalibration would predict adaptation to wheelchair locomotion would influence subsequent walking. Given the equivocal results from Experiment 3, it is likely that this influence would be small providing some support for recalibration of the different locomotion modality but that is weaker than recalibration of the matched modality. A limb-specific account of recalibration would predict no influence of wheelchair recalibration on subsequent walking.

### Methods

#### Participants

A total of 20 participants were randomly assigned to either a visually slower (*n* = 10) or visually faster (*n* = 10) recalibration condition.

#### Materials and Design

The same targets, target distances, noise-canceling equipment, blindfold, wheelchair, and real and virtual environments used in the previous experiments were employed in Experiment 4.

#### Procedure

Experiment 4 employed walking in pretest and posttest, but the adaptation phase consisted of wheeling through the virtual hallway while viewing rates of visual flow that were either faster or slower than wheeling speed. A preliminary experiment using the same blind-walking and wheelchair locomotion practice procedures used in Experiment 3 yielded no significant effect of recalibration of wheeling on subsequent blind-walking. As a result, participants in Experiment 4 were given more extensive wheelchair practice prior to beginning the pretest. The wheelchair practice was doubled to approximately 20 minutes of eyes open and eyes-closed wheeling (while spatial updating), followed by several minutes of practice walking with eyes closed.

During pretest, following this extended wheelchair practice, participants performed 10 blind-walking to target trials, as described in Experiment 1. Unlike Experiment 1, however, the intervention phase involved *wheeling* in the virtual hallway while viewing a rate of visual flow that was either twice or half wheeling speed. Following the 15th and final trial of the adaptation phase, participants were blindfolded, helped out of the wheelchair, and led back to the adjacent hallway to complete the posttest. During posttest, participants completed nine more trials of blind-walking to targets, using the same randomized target and distance order employed in the pretest.

### Results

Participants who experienced visual flow that was slower than wheeling speed undershot target distances by 0.07% (*SD*  = 3.29) in the posttest relative to the pretest. Participants who experienced visual flow that was faster than their wheeling speed undershot target distances by 2.82% (*SD*  = 7.61) in the posttest compared to pretest (see Figure 2).

A 2 (testing session: pretest or posttest) ×3 (distance) ×2 (visual speed condition) repeated measures ANOVA indicated only a significant main effect of target distance, *F*(2, 36)  = 47.26, *p*<.001, η_p_^2^  = .72. Across both conditions, pretest blind-walking was generally accurate at each of the three target distances (see Table 1). Unlike Experiment 3, an independent samples *t*-test showed that the difference between percent change from pre- to post-test across the two visual speed conditions was not significant, *t*(18)  = 1.05, *p* = .31.

**Figure 6:**
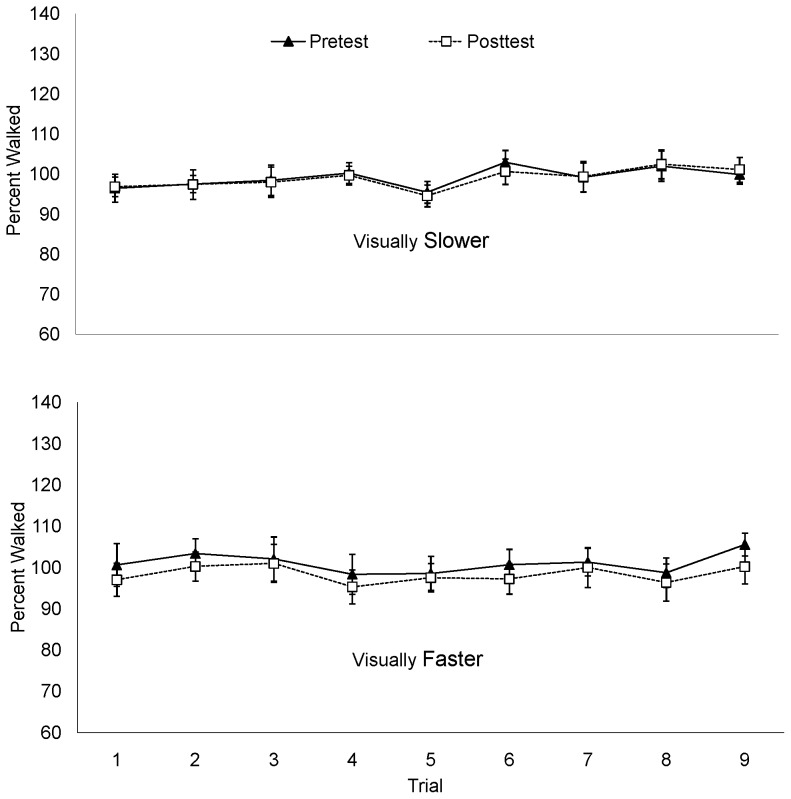
Percent walked by trial in Experiment 4.

The mean percent of target distance walked is plotted by trial in Figure 6. As seen in blind-walking in Experiment 1 (and in contrast to the distances wheeled in Experiments 2 and 3), there was no correlation between trial number and distance walked (p = .114). In other words, there was no evidence of a practice effect on distances walked. The distance walked in the first posttest trial of the visually-slower condition decreased 2.29% compared to mean pretest percent walked, while the distance walked in the first posttest trial of the visually-faster condition decreased 4.01% compared to the mean pretest percent walked. Paired samples t-tests for each condition showed that these differences were not significant (*p* = .16 for visually faster; *p* = .32 for visually slower). This percent change did not differ between conditions, as revealed by an independent samples t-test (*p = *.63).

### Discussion

There was no evidence of recalibration of wheelchair locomotion on subsequent blind-walking performance. Thus, support for generalization of recalibration mechanisms in locomotion broadly serving functionally similar goal of translation is not supported. As previously suggested, the lack of an effect of an effect of recalibration of wheelchair locomotion on subsequent walking may be due in part to participants’ inexperience with wheelchair locomotion. If participants were not initially well-calibrated to wheelchair locomotion, perhaps the discrepant visual-motor pairing during the intervention was not salient. However, the reasonable accuracy of wheeling in the pretests of Experiments 2 and 3 argues that participants were calibrated enough to show systematic effects of target distance with this mode of locomotion. Moreover, as previously mentioned, an initial experiment that included wheelchair practice that mirrored the practice sessions in Experiments 2 and 3 also failed to show a significant effect of recalibration of wheeling on subsequent walking. Even after more extensive practice prior to the experiment session as carried out in Experiment 4, it appears that participants did not generalize the calibration from wheelchair locomotion to walking. In light of the small effect in Experiment 3, we suggest that the generalizability of visual-motor calibration may be modulated by the similarity or familiarity of the locomotor action calibrated.

## General Discussion

In summary, recalibration of walking influenced subsequent blind-walking and the recalibration of wheeling influenced subsequent blind-wheeling. The recalibration effect found in the matched modality wheeling experiment is notable in itself, as it is a novel finding of a strong perceptual-motor recalibration effect shown with an unfamiliar mode of locomotion. Recalibration of walking only weakly influenced subsequent wheeling, suggesting that the perceptual-motor relationship learned in one locomotion modality may be just one of several sources of information influencing open-loop locomotion via other motor means. The lack of effect of wheeling adaptation on subsequent walking further illustrates the limits of generalization of perceptual-motor calibration. The strongest effects resulted when the same mode of locomotion was used in the adaptation and updating phases of the experiment. The weakest effect resulted in the transfer of the wheeling adaptation – an unfamiliar action – to walking. Together, these results modify the claim that actions that are functionally similar rely on the same perceptual-motor calibration.

There are several possible explanations for the weak effect of walking calibration on subsequent wheeling and the lack of an effect of recalibration of wheeling on subsequent walking. First, there are numerous ways of characterizing the functional similarity of actions and it may be the case that walking and wheeling are similar in only the broadest sense [11]. For example, there is evidence to suggest that calibration of walking influences biomechanically different actions that serve to move one through space, such as side-stepping [4] or crawling [13]. Both of these actions, unlike wheelchair locomotion, involve the application of thrust directly to the ground via the limbs in order to propel the body forward. If, during walking, the relationship between the application of thrust to the ground and visual indicators of self-motion is recalibrated, wheelchair locomotion may be unaffected because it involves a different motor action [11]. In this case, the perceptual-motor organization is not limb-specific or motor-specific, but is tied to the relationship between motor movement’s interface with the environment and the corresponding perceptual changes (e.g. visual flow). By this account, recalibration of walking would influence walking on the hands, but not wheeling or pedaling a bicycle or any other form of locomotion that does not involve producing thrust directly against the ground plane. Alternatively, it is possible that the weak influence of recalibration of walking on wheeling and the absence of an effect of recalibration of wheeling on walking indicate at least a partially limb-specific organization of perceptual-motor relationships. Research investigating adaptation to visual field shifts while wearing wedge prism glasses suggests that such adaptation is highly specific to the adapted limb and even the adapted motion executed by that limb [22], [23], [24]. However, Morton and Bastian [25] illustrated that adaptation to lateral displacement from wearing prisms while walking led to an aftereffect in a reaching task. Generalization from one movement task to another was asymmetric: adaptation during the reaching task did not influence subsequent walking (but see [26] for the opposite finding). It is possible that the present results may be seen as analogous to the results from these studies in that wheelchair movements that rely on movements of the upper extremities (and could be construed as a reaching task) do not generalize to locomotion that involves the lower extremities, but walking generalizes more broadly. There is, however, an important distinction between adaptation resulting from lateral displacement using prisms and the present studies that manipulated gain during locomotion. Prism adaptation is thought to involve two components: an initial error-correction and a more gradual spatial realignment of visual and proprioceptive spatial maps [27]. During prism adaptation, movement errors are detectable (i.e. initially, movement towards a target results in a clear discrepancy between movement endpoint and target location). In the present experiments, adaptation involved no such error-corrective feedback regarding movement accuracy – participants walked (without a target or goal destination) while experiencing a discrepant rate of visual flow. Anecdotally, most participants reported noticing nothing unusual about the rate of visual flow during the adaptation phase of the experiment. However, even if participants had noticed a discrepancy between the visual flow and biomechanical locomotion speed, there was no way to correct this discrepancy through their actions. The experiments described here also differ from prism adaptation experiments in that adaptation during throwing or reaching while wearing prism involves correcting for actions performed based on altered stimuli. In visual-motor recalibration, the adapted behavior is manifested in a task in which there is no altered stimuli.

It is also interesting to consider why a small influence of recalibration of walking on wheelchair locomotion might occur. Given humans’ typical extensive experience with walking, it is likely that sources of information about the rate of self-motion that are tied to walking are weighted more heavily than other sources of information for the calibration of locomotion. Thus, a new perceptual-motor calibration formed during the familiar act of walking may be more likely to influence a less well-established form of locomotion such as wheeling. In the context of sensory integration, optimal-integration of cues has been studied in visual-haptic integration in judging shape, position, or other object dimensions [28], visual cues for depth [29], [30], and sensorimotor learning and rate of adaptation [31], [32]. Bayesian theories of sensory integration suggest that, under conditions of uncertainty, prior knowledge tends to more strongly influence a system's decision-making than the degraded available cues. Probability distributions (or prior knowledge) for visual-motor pairings involved in walking are likely to be better established than those for wheelchair locomotion. Why then, does wheeling rely more strongly on visual-motor calibration of wheeling rather than walking? There is evidence that task demands play a role in the weighting of cues [33] and, in the context of visual-proprioceptive conflict, that visual and proprioceptive cues are weighted based on knowledge about their precision [34], [35]. Just as vision tends to dominate proprioception for localizing limb position in cases of conflict [36], perceptual-motor calibration of walking may serve as the most salient source of information about spatial orientation during self-movement. However, task demands of wheeling may shift the weighting of cues to utilize the calibration of wheeling, much like hand movements in azimuthal directions rely more on proprioceptive cues than visual cues [34], [35].

For novice wheelchair users, the effect of recalibration of wheeling may be more limited in terms of generalizability across forms of locomotion, but with a greater magnitude effect on biomechanically similar locomotion, as suggested by Experiment 2. Experiment 4 suggests that wheelchair training would likely need to be extensive to make recalibration symmetrical for walking and wheelchair locomotion. Others have shown that novice wheelchair users are inaccurate at judging their ability to wheel through apertures, even after wheelchair training [37] (but see also [38]). Similarly, younger wheelchair users with cerebral palsy are less accurate at judging apertures through which they can wheel than older wheelchair users [39]. Future studies with experienced wheelchair users could determine whether calibration of wheeling has more robust effects on subsequent walking given expertise with the perceptual-motor relationships involved in wheeling. Other non-walking forms of self-movement are likely to be informed by perceptual-motor calibration of walking, but the influence may be mediated by the biomechanical similarity to walking and/or one’s familiarity with the action.

In the present research, the biomechanical dissimilarity between walking and wheeling was confounded with the familiarity (or lack thereof) of each form of locomotion. Future work should employ forms of locomotion that are biomechanically distinct from walking but that are highly practiced or familiar or should examine the possible transfer of recalibration from one unfamiliar form of locomotion to another equally novel form of locomotion.

### Conclusions

From a theoretical perspective, this work more precisely defines the role of calibration of locomotion in maintaining spatial orientation. Knowing where we are in the environment (i.e., spatial orientation) is likely to be informed by perceptual-motor calibration, but the extent to which this knowledge is informed by our prior locomotor experiences is largely unknown. The experiments described here suggest that dynamic spatial orientation is influenced by the learned relationship between perceptual and motor information for self-motion. This generalizes to locomotion modalities that are less experienced, as seen in wheelchair locomotion, but only strongly when the adaptation and response modalities match.

Furthermore, the present results provide insights into the perceptual-motor relationships involved in methods used to probe spatial perception. The use of visually-directed actions such as blind-walking is now commonplace in spatial perception research, but there are few studies that have assessed the importance of establishing and maintaining calibration during blind-walking. Philbeck and colleagues demonstrated a tendency to overshoot targets over repeated blind-walking trials – a finding that may be attributable to perceptual-motor recalibration of walking over successive walking without vision trials [40]. Clearly, for experiments that rely on blind-walking or spatial updating, it is important to understand and account for the influence of prior visual-motor experience/calibration.

From a practical perspective, more complete knowledge of the processes and generalizability of perceptual-motor calibration of walking will contribute to an understanding of perceptual and motor learning and may lead to improvements in training and rehabilitation. For example, consider the challenges of adapting to the use of wheelchair or prosthetic limb. Learning to navigate using unfamiliar means of locomotion requires, in part, calibrating perceptual-motor systems. The present work highlights the importance of calibration for wheelchair locomotion and suggests that novel locomotor calibrations are not easily transferred to other means of locomotion.

The results may also contribute to theories of perceptual-motor skill learning in contexts other than locomotion. For example, Fajen [41] has framed learning to brake to avoid collisions and to rapidly adjust braking behaviors in the context of perceptual-motor calibration More generally, Fajen [42] has described several other visually-guided actions such as intercepting a target and steering in the context of continuous calibration between visual information and the body’s actions or potential for action. It is likely that many types of perceptual-motor skill learning involve initial and ongoing calibration. The current results shed some light on the influence of a given form of perceptual-motor calibration on related, but motorically distinct actions. The generalizability of calibration/recalibration of actions other than walking and rotating is largely unexplored.

Perceptual-motor calibration has rarely been explored in developmental or applied domains, but the current results can shed light on the role of calibration in training and rehabilitation. Adolph and Avolio [43] demonstrated the adaptability of infants to changing body characteristics by adding weights to infant’s legs and observing their willingness to walk down a slope. It is likely that calibration over a longer time scale allows for growing children to adapt to changing body dimensions. Long-term calibration of a given actions such as walking may impact learning of a variety of forms of movement and actions. Astronauts are trained in simulated low-gravity situations in order to calibrate visual-motor systems in preparation for space flight. Mulavara and colleagues [44] investigated the recovery time for astronauts returning from extensive space flight and showed that full motor function recovery could take up to 15 days. They also showed that recovery followed the adaptive course previously described with initial strategic adaptation of motor responses, followed by a more gradual perceptual relearning. The generalizability of recalibration described here may eventually lead to training/retraining methods that will minimize training time with maximum impact across different types of actions.
